# Sexual behaviors and seroprevalence of HIV, HBV, and HCV among hill tribe youths of Northern Thailand

**DOI:** 10.1186/s12889-019-7459-9

**Published:** 2019-08-14

**Authors:** Tawatchai Apidechkul

**Affiliations:** 1 Center of Excellence for the Hill tribe Health Research, Mae Fah Laung University, Chiang Rai, Thailand; 2 School of Health Science, Mae Fah Luang University, Chiang Rai, Thailand

**Keywords:** Sexual behaviors, HIV, HBV, HCV, Seroprevalence, Hill tribe, Youths

## Abstract

**Background:**

Sexual behaviors reflect the degree of exposure to human immunodeficiency virus (HIV), hepatitis B virus (HBV), and hepatitis C virus (HCV), especially in people in sexually active stages, such as youths. Hill tribe people have their own cultures, beliefs and lifestyles related to their behaviors, including sexual behaviors, which may lead to HIV, HBV, and HCV infections, especially among youths. The study aimed to examine sexual behaviors and assess the seroprevalence of HIV, HBV, and HCV among hill tribe youths.

**Methods:**

A cross-sectional study was conducted. The participants were recruited from 60 randomly selected hill tribe villages in Chiang Rai Province, Thailand. A validated questionnaire and 5 mL blood specimens were used to collect data. Data were collected by a self-reporting method. Rapid immunochromatographic tests were used to detect hepatitis B surface antibody (anti-HBs), hepatitis B surface antigen (HBsAg), hepatitis C antibody (anti-HCV), and human immunodeficiency virus antibody-I and- II (anti-HIV-1 and -2). Chi-square and Fisher’s exact test were used to detect the associations between variables.

**Results:**

A total of 1325 participants were recruited for the analysis. The majority were females (60.5%) and aged 15–17 years (58.9%). A total of 14.5% smoked, 22.4% drank alcohol, 14.2% were tattooed, and 61.4% had their ears pierced. Among the 30.3% who had sexual experience, 42.0% experienced one-night stands, 26.9% had sexual contact with a prostitute within 1 year prior to the study, 18.9% used alcohol prior to having sexual intercourse, and 15.7% had been tested for HIV/AIDS previously. Among males, 11.5% were males who had sex with males (MSM), and 4.6% were bisexual. Among females, 83.0% were females who had sex with males, and 5.0% were females who had sex with females. Different sexes and tribes were found to have significantly different risk behaviors and sexual behaviors, such as overall males having a greater proportion of sexual experience than females, and Lahu, Akha and Hmong had a higher proportion of sexual experience, having sexual experience with one-night stands, and having sexual experience with a prostitute 1 year prior to the study than others. Among the 836 obtained blood samples, none were positive for anti-HIV-I and -II, 6.4% were positive for anti-HBs, 1.9% were positive for HBsAg, and 0.2% were positive for anti-HCV.

**Conclusion:**

Hill tribe youths in Thailand are at risk of STIs such as HBV and HCV infections according to their risk behaviors and sexual behaviors, which differ between sexes and tribes. Effective behavioral interventions should be promoted among hill tribe youths to minimize the risk for these diseases in the future.

## Background

Individual sexual behaviors reflect the degree of risk for sexually transmitted infections (STIs) [1], which are caused by more than 30 different bacteria, viruses and parasites known to be transmitted through sexual contact, particularly human immunodeficiency virus (HIV), hepatitis B virus (HBV), and hepatitis C virus (HCV), which are identified as major human health threats today [2]. Expressions of sexual behaviors are influenced by both internal and external factors [3–5]. Internal factors include age, sex, hormones, etc., which stimulate the interest in or expression of sexual activities in humans [4, 5]. Sexual behaviors also depend on the stage of physical and mental development [6, 7]. Several studies have reported that sociocultural factors act as external factors to express or suppress some human-related sexual behaviors [8–10]. Some communities widely accept sexual-related behaviors in public areas, while others condemn this behavior [11]. Some communities accept polygamy, while others do not [12]. Sex workers are legal and allowed in some countries, whereas they are not allowed in others [13, 14]. However, reproduction and genetic continuation are the major factors driving sexual behaviors in all species, including humans [15].

Different communities have different levels of social acceptance or suppression of the expression of their sexual behaviors [16, 17]. Sexual behaviors in different communities or groups of people are also dominated by their economy, culture, lifestyle, etc. [18]. Moreover, the patterns or preferences of sexual behaviors are also different from person to person and community to community, including people living in Thailand [19–21]. Many communities have already accepted the differences in human sexual behaviors or sexual patterns, such as male-to-female sexual intercourse and male-to-male sexual intercourse. [22, 23]. In addition, sexual behaviors are influenced by other factors, such as alcohol, drugs, the media, etc. [5, 8, 19, 24]. The use of alcohol or substances in different groups or different communities of people depends on various factors, including personal characteristics, education level, social status, culture, income, etc. [19, 20, 24]. Different sexual behaviors lead to different levels of exposure to HIV, HBV, and HCV [25, 26]. The United Nation defines that those people aged 15–24 are youths [27]. Youths are also defined as the sexually active stage [28]. Therefore, from several studies mentioned above [3–5, 8–10, 19, 24], youths aged 15–24 years are at the sexually active stage of life; as a result, they become one of the most vulnerable populations for drug and alcohol use and, consequently, at high risk of HIV, HBV, and HCV infections.

In 2017, the World Health Organization (WHO) reported that HIV/AIDS, HBV, and HCV were major global communicable diseases for humankind in this era with a large population of infected persons. Approximately 35 million are HIV-infected [29], 257 million are HBV-infected [30], and 71 million have chronic hepatitis C infections globally [31]. Currently, these diseases are widely recognized as major public health problems worldwide with significant impacts on human health and human life, particularly among those people who are living in highly epidemic areas, including Thailand [29].

Thailand has one of the highest HIV, HBV and HCV burdens in the world [32, 33], particularly among those with low socioeconomic status, including the hill tribe people [32]. Hill tribes are a group of people who have their own cultures, beliefs and lifestyles [34]. They migrated from the south of China and have settled in the border regions of Thailand-Myanmar-Laos over the past centuries [34]. In 2017, approximately 3.5 million hill tribe people lived in Thailand, and 300,000 hill tribe people lived in Chiang Rai Province [35] located in the northernmost part of Thailand. They are classified into six main tribes: Akha, Lahu, Hmong, Yao, Lisu, and Karen [35]. Each tribe has their own culture, lifestyle, and level of acceptance for the use of alcohol and substances in their communities [36]. Most hill tribe people are of low socioeconomic status, and some of them do not possess Thai identification cards (ID cards), which are used for free access to all public services, including education and health care systems [37]. Today, the hill tribe people who live in Thailand are the second and third generations of the first hill tribe generation that had moved to and settled in Northern Thailand in the early twentieth century [34]. Many hill tribe people still practice their own traditions, cultures and lifestyles, including sexual behaviors, particularly sexual intercourse in their early age, use alcohol before having sex, etc. [37]. Some tribes accept monogamy, and others accept polygamy. Akha accepts polygamy, while Lahu accepts getting married in younger age, less than 15 years [38]. Under globalization and urbanization, many have changed their sexual behaviors. They often work with or are exposed to people outside their community and have integrated with a new society with advanced communication technology, population migration, rapid economic growth, and a mixture of cultures, resulting in a risk of HIV, HBV, and HCV infection. Moreover, many factors may influence their sexual behaviors and practices, particularly among those in the sexually active stage of life and those aged 15–24 years.

There is limited scientific information available on sexual behaviors and HIV, HBV, and HCV among hill tribe youths. Therefore, this study aimed to understand the context of sexual behaviors and estimate the prevalence of HIV, HBV, and HCV among hill tribe youths aged 15–24 years. The study also aimed to assess several risk behaviors that could be associated with STIs between sexes and tribes among hill tribe youths.

## Methods

### Study design

A cross-sectional study was conducted to elicit information from the participants.

### Study setting

In 2016, there were 749 hill tribe villages in Chiang Rai, which included 316 Lahu villages, 243 Akha villages, 63 Yao villages, 56 Hmong villages, 36 Karen villages, and 35 Lisu villages. In 2016, a total of 41,366 hill tribe families lived in Chiang Rai Province [35].

The 60 hill tribe villages in Chiang Rai, Thailand were the study setting. Ten villages of each tribe were randomly selected from a list of hill tribe villages based on data from 2017.

### Study population

The study populations were hill tribe youths aged 15–24 years.

### Inclusion and exclusion criteria

Inclusion criteria include being a hill tribe youth from one of the six hill tribe populations, aged 15–24 years old at the date of data collection, and fluent in Thai. Those selected samples who had a physical or mental condition resulting in the inability to provide essential information regarding the study protocols were excluded from the study.

### Sample size

The sample size was calculated based on the following formula, which was developed by Daniel in 1999 [39], to find the minimum number for the study that was used while we did not know a certain number of the study population. The sample size was calculated based on the prevalence from previous or pilot studies.

$$ \mathrm{n}=\left[{{\mathrm{Z}}^2}_{\upalpha /2}\mathrm{PQ}\right]/{\mathrm{e}}^2 $$

Where n is the sample size required. Z was set at 1.96. P was assumed to be 0.17, which was the prevalence of HBsAg based on a study by Pichainarong et al. [40], which was a very important number for obtaining the best precision of the findings; then, the Q was 0.83. The accepted error or type one error in this study was set at e = 0.05, and the confidence interval was 95%. At least 1301 participants were required for the analysis. Then, we used the same number of participants in all six tribes.

### Research instruments

A questionnaire and 5 mL blood specimens were used for data collection. A questionnaire was developed from a literature review, and an in-depth interview with 12 selected participants (six men and six women) aimed to ensure that all relevant information regarding sexual behaviors of the hill tribe youths was included in the questionnaire. It was then tested for validity using the item-objective congruence (IOC) technique, which was performed by three external experts in relevant fields. A pilot study was conducted with 20 selected samples (ten men and ten women) who had similar characteristics to the study participants to assess the reliability and feasibility of the questionnaire.

The questionnaire consisted of six parts. The first part consisted of 13 questions used to collect the participants’ general information, such as sex, age, tribe, marital status, income (referred to the money getting from work, not included having daily or weekly allowance from parents), etc. The second part consisted of 10 questions used to collect the participants’ health behaviors, such as smoking, alcohol use, and methamphetamine use. Three answer choices were provided for each question: yes, no, and quit using it. The third part consisted of 15 questions used to collect information on the participants’ sexual behaviors such “Do you have a partner?”, “Have you had sexual experience?”, “Did you use condom while having sex?”, “How many partners?”, “Have you ever been tested for HIV?”, “Have you been tested for HIV in the past 6 months? (from the interview date)”, etc.

The fourth part consisted of 8 questions used to collect information on the history of STIs, such as “Did you experience genital discharge?”, “Did you have itching in your genital organs?”, “Did you have bad smell from your genitals?”, etc. For these questions, the two answer choices were “yes” and “no”.

The fifth part contained 6 questions that were used to collect information regarding their experiences in receiving information regarding STIs and sources of information. The last part consisted of questions used to detect the knowledge and attitude of risk of STIs. For the knowledge questions, the answers were “correct” “not sure”, and “incorrect”. For the attitude section answers, the answers were “agree” “neutral” and “disagree”. In the last two parts of the questionnaire, there were questions in relation to the experience of information regarding STIs and the knowledge and attitude of risk of STIs. However, after analysis, there was no outcome of interest from questions part five and six; then, they were omitted from the results.

## Laboratory analysis

Rapid immunochromatographic tests were used to detect hepatitis B surface antibody (anti-HBs), hepatitis B surface antigen (HBsAg), hepatitis C antibody (anti-HCV), and human immunodeficiency virus antibody-1 and-2 (anti-HIV-1 and -2). The Wondfo Diagnostic Kit® was used to detect anti-HBs, which showed a sensitivity of 97.3% and a specificity of 99.2%. The Wondfo One Step HBsAg Serum/Plasma Test® was used for HBsAg detection, which showed a sensitivity of 96.2% and a specificity of 99.3%. The Quick Hepatitis C Virus Serum/Plasma Test was used to detect antibodies to HCV, which showed a sensitivity of 96.6% and a specificity of 99.5%. Finally, the Wondfo Diagnostic Kit for HIV-1 and -2 Antibodies® was used to detect the antibodies of HIV-1 and HIV-2, with a sensitivity of 100.0%.

### Process of data gathering

Access to villages was granted by district government officers. All selected village headmen were contacted 2 days before approaching the participants for data collection. All youths who were living in the selected villages were listed by the village headman. Those who met the inclusion and exclusion criteria were invited to participate in the study and were informed by the village headman regarding the research objectives and protocols through the village broadcast system.

Upon reaching the village, participants were provided all essential information, and informed consent was obtained on a voluntary basis form before completing the questionnaire by a self-administered method in a private and confidential room at the village. Five milliliter blood specimens were collected. Blood specimens were packed and kept in a temperature-controlled box and were delivered to the laboratory for analysis the same day at the Mae Fah Laung University Medical Laboratory Center.

### Statistical analysis

Data were double entered into an Excel sheet. Before analysis, the data were cleaned. No missing data were found. Data were analyzed by SPSS version 24, 2016 (SPSS, Chicago, IL). Descriptive and inferential statistics were used for analysis and interpretation of the results. A chi-square test or Fisher’s exact test was used to detect the associations between variables at the alpha = 0.05 significance level.

### Ethical consideration

All research proposals, protocols and consents to participate were approved by the Mae Fah Luang University Research Ethics Committee on Human Research (REH-60030). Interviews for data collection and blood specimen collections for laboratory testing were conducted after all participants received oral and written explanations regarding the study objectives and procedures, including any possible risks for physical and mental harm; participants voluntarily agreed by signing on the consent form. Parental consent was obtained from the parent/guardian of participants aged less than 18 years after given the explanations by a village headman and their child (participants). After completion of the analysis, all completed questionnaires were destroyed properly. All relevant data files were kept on a personal computer with secure passwords. A small gift (a piece of towel that was less than 2$US) was provided to all participants to show appreciation for their cooperation.

## Results

In total, 1325 participants were enrolled in the study. The majority were females (60.5%), aged 15–17 years (58.9%), and single (94.3%). More than half were Buddhist (56.1%) and lived with their parents (59.8%). A total of 72.5% graduated high school, and 90.0% had no income (Table 1).

**Table 1:** Demographic characteristics of the participants

Characteristics	n (%)
Total	1325 (100.0)
Sex	
Male	524 (39.5)
Female	801 (60.5)
Age (year)	
15–17	780 (58.9)
18–20	398 (30.0)
21–24	147 (11.1)
Tribe	
Akha	449 (33.9)
Lahu	207 (15.6)
Hmong	215 (16.2)
Yao	177 (13.4)
Karen	160 (12.1)
Lisu	117 (8.8)
Marital status	
Single	1250 (94.2)
Married	65 (5.0)
Separated	10 (0.8)
Religion	
Buddhism	743 (56.1)
Christianity	574 (43.3)
Islam	8 (0.6)
Education	
Illiterate	47 (3.5)
Primary school	52 (3.9)
High school	960 (72.5)
Vocational school	248 (18.7)
University	18 (1.4)
Occupation	
Unemployed	114 (8.6)
Student	1058 (79.8)
Farmer	39 (2.9)
Laborer	104 (7.8)
Merchant	10 (0.8)
Income	
No	1192 (90.0)
Yes	133 (10.0)
Living place	
Own house	935 (70.6)
Dormitory	331 (25.0)
Relative’s house	51 (3.8)
Other	8 (0.6)
Living with	
Alone	97 (7.3)
Parents	793 (59.8)
Father	31 (2.3)
Mother	98 (7.4)
Relative	106 (8.0)
Brother	59 (4.5)
Friends	98 (7.4)
Partners	41 (3.1)
Employer	2 (0.2)

Participants used different substances in different proportions: 14.5% smoked, 22.4% drank alcohol, 2.6% used methamphetamines, and 4.5% used marijuana. In addition, 14.2% had tattoos, 61.4% had ear piercings, 12.2% had a history of medical surgery, 4.0% had experienced injection from illegal practitioners, and 2.0% had experienced acupuncture (Table 2).

**Table 2:** Comparison of risk behaviors between the sexes

Characteristics	Total n	%	Male		Female		χ^2^	*p*-value
			n	%	n	%		
Total	1325	100.0	524	39.5	801	60.5	N/A	N/A
Smoking								
Yes	192	14.5	165	85.9	27	14.1	202.12	< 0.001^*^
No	1133	85.5	359	31.7	774	68.3		
Alcohol use								
Yes	297	22.4	205	69.0	92	31.0	139.12	< 0.001^*^
No	1028	77.6	319	31.0	709	69.0		
Methamphetamine use								
Yes	35	2.6	29	82.9	6	17.1	28.20	< 0.001^*^
No	1290	97.4	495	38.4	795	61.6		
Heroin use								
Yes	23	1.8	19	82.6	4	17.4	18.15	< 0.001^*^
No	1302	98.2	505	38.8	797	61.2		
Crystal methamphetamine use								
Yes	20	1.5	15	75.0	5	25.0	10.67	0.001^*^
No	1305	98.5	509	39.0	796	61.0		
Opium use								
Yes	21	1.7	17	81.0	4	19.0	15.30	< 0.001^*^
No	1304	98.4	507	38.9	797	61.1		
Marijuana use								
Yes	60	4.5	54	90.0	6	10.0	66.91	< 0.001^*^
No	1265	95.5	470	37.2	795	62.8		
Tattooed								
Yes	188	14.2	119	63.3	69	36.7	51.69	< 0.001^*^
No	1137	85.8	405	35.6	732	64.4		
Ear piercing								
Yes	813	61.4	193	23.7	620	76.3	219.91	< 0.001^*^
No	512	38.6	331	64.6	181	35.4		
History of blood transfusion								
Yes	50	3.8	20	40.0	30	60.0	0.004	0.947
No	1275	96.2	504	39.5	771	60.5		
History of organ transplant								
Yes	11	0.8	8	72.7	3	27.3	5.10	0.024^*, a^
No	1314	99.2	516	39.3	798	60.7		
History of medical surgery								
Yes	161	12.2	81	50.3	80	49.7	8.88	0.003^*^
No	1164	87.8	443	38.1	721	61.9		
Injection from illegal practitioners								
Yes	53	4.0	33	62.3	20	37.7	11.91	0.001^*^
No	1272	96.0	491	38.6	781	61.4		
Acupuncture								
Yes	26	2.0	16	61.5	10	38.5	5.36	0.021^*^
No	1299	98.0	508	391	791	60.9		
Shared a toothbrush								
Yes	267	20.2	99	37.1	168	62.9	0.85	0.356
No	1058	79.8	425	40.2	633	59.8		
History of hepatitis B vaccination								
Yes	116	8.8	42	63.2	74	63.8	3.17	0.366
No	476	35.9	192	40.3	284	59.7		
Not sure	433	32.7	161	37.2	272	62.8		
Unknown	300	22.6	129	43.0	171	57.0		
Family history of hepatitis B								
Yes	31	2.3	12	38.7	19	61.3	0.79	0.850
No	797	60.2	315	39.5	482	60.5		
Not sure	185	14.0	78	42.2	107	57.8		
Unknown	312	23.5	119	38.1	193	61.9		
Work experience outside the village								
Yes	143	17.1	82	57.3	61	42.7	29.56	< 0.001^*^
No	693	82.9	230	33.2	463	66.8		
Work experience abroad								
Yes	10	1.2	4	40.0	6	60.0	0.31	0.860^a^
No	826	98.8	308	37.3	518	62.7		

^*^Significance level at α =0.05

^a^Fisher’s exact test

While making comparisons between sexes regarding risky behaviors, 14 behaviors or variables were significantly different between the sexes: smoking, alcohol use, methamphetamine use, heroin use, crystal methamphetamine use, opium use, marijuana use, tattoos, ear piercings, a history of organ transplants, a history of medical surgery, a history of drug injection from illegal practitioners, acupuncture, and experience working outside the village. The male sex made up a greater proportion of all variables except ear piercing, which was greater in females (Table 2).

In total, 402 persons (30.3%) reported having sexual experience (average age of first sexual intercourse was 16.7 years, with a minimum of 12 years and maximum of 23 years). Both males and females had similar proportions of having girlfriends (38.5%) and boyfriends (38.2%). 80.6% reported having sexual experience with their current boyfriend or girlfriend. Among those who had sexual experience, 42.0% had experienced one-night stands, 26.9% had sexual experience with prostitutes (commercial sex workers) within 1 year prior to the study, 36.0% had regular sexual partners, and 18.9% used alcohol prior to having sexual intercourse. Sixty-three (15.7%) participants had previously experienced testing for HIV/AIDS. However, only 5.0% had been tested for HIV/AIDS within the 6 months prior to the study (Table 3).

**Table 3:** Comparison of sexual behaviors between sexes

Characteristics	n	%	Male		Female		χ^2^	*p*-value
			n	%	n	%		
Having boyfriend/girlfriend								
Yes	509	38.4	202	39.7	307	60.3	0.01	0.935
No	816	61.6	322	39.5	494	60.5		
Number of boyfriends/girlfriends								
Only one	489	96.1	187	38.2	302	61.8	10.84	0.001^*^
More than one	20	3.9	15	75.0	5	25.0		
Sexual experience								
Yes	402	30.3	194	48.3	208	51.7	18.31	< 0.001^*^
No	923	69.7	330	35.8	593	64.2	28.76	
Sexual experience with current boyfriend/girlfriend								
Yes	324	80.6	141	43.5	183	56.5	15.02	< 0.001^*^
No	78	19.4	53	67.9	25	32.1		
Sexual experience with one-night stand								
Yes	169	42.0	99	58.6	70	41.4	12.43	< 0.001^*^
No	233	58.0	95	40.8	138	59.2		
Sexual experience with a prostitute 1 year prior to the study								
Yes	108	26.9	61	56.5	47	43.5	3.99	0.046^*^
No	294	73.1	133	45.2	161	54.7		
Having a regular partner								
Yes	145	36.0	50	34.5	95	65.5	17.23	< 0.001^*^
No	257	64.0	144	56.0	113	44.0		
Use alcohol or drugs prior to having sexual intercourse								
Yes	76	18.9	40	52.6	36	47.4	1.24	0.536
Sometimes	18	4.5	10	55.6	8	44.4		
No	308	76.6	144	46.8	164	53.2		
HIV testing experience								
Yes	63	15.7	30	47.6	33	52.4	0.01	0.912
No	339	84.3	164	48.4	175	51.6		
HIV testing 6 months prior to the study								
Yes	43	5.0	23	53.5	20	46.5	0.52	0.468
No	359	73.1	171	47.6	188	52.4		
HIV testing 6 months to 1 year prior to the study								
Yes	41	10.2	22	53.7	19	46.3	0.53	0.465
No	361	89.8	172	47.6	189	52.4		

^*^Significance level at α =0.05

In the comparison of sexual behaviors between males and females, six variables were found to be significantly different between sexes: number of boyfriends or girlfriends, sexual experience, sexual experience with current boyfriends or girlfriends, sexual experience with one-night stands, sexual experience with a prostitute within 1 year prior to the study, and having a regular partner. These variables were found to be more common among boys than girls. However, there were no significant differences between sexes regarding testing for HIV (Table 3).

Regarding the risk behaviors between the tribes, four variables were statistically significant: smoking, alcohol use, hepatitis B vaccination in family members, and work experience outside the village (Table 4). However, some variables could not be analyzed due to the small sample size in cells.

**Table 4:** Comparison of risk behaviors among tribes

Characteristics	Akha		Lahu		Hmong		Yao		Karen		Lisu		χ^**2**^	*p*-value
	n	%	n	%	n	%	n	%	n	%	n	%		
Total	449	33.9	207	15.6	215	16.2	177	13.4	160	12.1	117	8.8	N/A	N/A
Smoking														
Yes	64	33.3	44	22.9	20	10.4	14	7.3	28	14.6	22	11.5	21.45	0.001*
No	385	34.0	163	14.4	195	17.2	163	14.4	132	11.7	95	8.4		
Alcohol use														
Yes	84	28.3	57	19.2	35	11.8	38	12.8	49	16.5	34	11.4	20.58	0.001*
No	365	35.5	180	17.5	180	17.5	139	13.5	111	10.8	83	8.1		
Methamphetamine use														
Yes	18	51.4	4	11.4	4	11.4	1	2.9	6	17.1	2	5.7	N/A	N/A
No	431	33.4	203	15.7	221	16.4	176	13.6	154	11.9	115	8.9		
Heroin use														
Yes	10	43.5	7	30.4	4	17.4	0	0.0	1	4.3	1	4.3	N/A	N/A
No	439	33.7	200	15.4	211	16.2	177	13.6	159	12.2	116	8.9		
Crystal Methamphetamine use														
Yes	10	50.0	4	20.0	3	15.0	0	0.0	2	10.0	1	5.0	N/A	N/A
No	439	33.6	203	15.6	212	16.2	177	13.6	158	12.1	116	8.9		
Opium use														
Yes	9	42.9	5	23.8	3	14.3	0	0.0	3	14.3	1	4.8	N/A	N/A
No	440	33.7	202	15.5	212	16.3	177	13.6	157	12.0	116	8.9		
Marijuana use														
Yes	25	41.7	8	13.3	6	10.0	10	16.7	9	15.0	2	3.3	5.90	0.306
No	424	33.5	199	15.7	209	16.5	167	13.2	151	11.9	115	9.1		
Tattooed														
Yes	74	39.4	36	19.1	23	12.2	24	12.8	16	8.5	15	8.0	8.37	0.137
No	375	33.0	171	15.0	192	16.9	153	13.5	144	12.7	102	9.0		
Ear piercing														
Yes	285	35.1	134	16.5	131	16.1	100	12.3	92	11.3	71	8.7	4.65	0.460
No	164	32.0	73	14.3	84	16.4	77	15.0	68	13.3	46	9.0		
History of blood transfusion														
Yes	18	36.0	12	24.0	5	10.0	1	2.0	8	16.0	6	12.0	N/A	N/A
No	431	33.8	195	15.3	210	16.5	176	13.8	152	11.9	111	8.7		
History of organ transplants														
Yes	6	54.5	4	36.4	0	0.0	0	0.0	1	9.1	0	0.0	N/A	N/A
No	443	33.7	203	15.4	215	16.4	117	13.5	159	12.1	117	8.9		
History of medical surgery														
Yes	48	29.8	30	18.6	27	16.8	16	9.9	20	12.4	20	12.4	6.29	0.278
No	401	34.5	177	15.2	188	16.2	161	13.8	140	12.0	97	8.3		
Injection from illegal practitioners														
Yes	23	43.4	12	22.6	3	5.7	5	9.4	3	5.7	7	13.2	10.72	0.057^a^
No	426	33.5	195	15.3	212	16.7	172	13.5	157	12.3	110	8.6		
Acupuncture														
Yes	6	23.1	8	30.8	4	15.4	1	3.8	3	11.5	4	15.4	7.91	0.161^a^
No	443	34.1	199	15.3	211	16.2	176	13.5	157	12.1	113	8.7		
Shared a toothbrush														
Yes	88	33.0	31	11.6	48	18.0	42	15.7	35	13.1	23	8.6	5.88	0.318
No	361	34.1	176	16.6	167	15.8	135	12.8	125	11.8	94	8.9		
History of hepatitis B vaccination														
Yes	44	37.9	12	10.0	19	16.4	16	13.8	14	12.1	11	9.5	16.02	0.381
No	174	36.6	71	14.9	70	14.7	67	14.1	52	10.9	42	8.8		
Not sure	136	31.4	66	15.2	81	18.7	48	11.1	60	13.9	42	9.7		
Unknown	95	31.7	58	19.3	45	15.0	46	15.3	34	11.3	22	7.3		
Family history of hepatitis B														
Yes	8	25.8	8	25.8	8	25.8	7	22.6	0	0.0	0	0.0	34.23	0.003*
No	3000	37.6	115	14.4	112	14.1	109	13.7	91	11.4	70	8.8		
Not sure	60	32.4	28	15.1	33	17.8	22	11.9	21	11.4	21	11.4		
Unknown	81	26.0	56	17.9	62	19.9	39	12.5	48	15.4	26	8.3		
Work experience outside the village														
Yes	50	35.0	29	30.3	13	9.1	14	9.8	25	17.5	12	8.4	17.94	0.003*
No	201	29.0	81	11.7	119	17.2	122	17.6	105	15.2	65	9.4		
Work experience abroad														
Yes	3	3.0	3	30.0	0	0.0	0	0.0	0	0.0	4	40.0	N/A	N/A
No	248	30.0	107	13.0	132	16.0	136	16.5	130	15.7	73	8.8		

*N/A* No statistical analysis available

^*^Significance level at α =0.05

^a^Fisher’s exact test

Regarding sexual behaviors between the tribes, six variables were found to have statistical significance: having a boyfriend/girlfriend, number of boyfriends or girlfriends, sexual experience, HIV testing experience, HIV testing within 6 months prior to the study, and HIV testing within 6 months to 1 year prior to the study. These variables of having a boyfriend/girlfriend, number of boyfriends or girlfriends, and sexual experience were found to be more common among the Akha, Lahu, and Hmong than other tribes (Table 5).

**Table 5:** Comparison of sexual behaviors between tribes

Characteristics	Akha		Lahu		Hmong		Yao		Karen		Lisu		χ^2^	*p*-value
	n	%	n	%	n	%	n	%	n	%	n	%		
Having boyfriend/girlfriend														
Yes	149	29.3	88	17.3	107	21.0	63	12.4	56	11.0	46	9.0	19.79	0.001*
No	300	36.8	119	14.6	108	13.2	114	14.0	104	12.7	71	8.7		
Number of boyfriends/girlfriends														
Only one	147	30.1	76	15.5	104	21.3	63	12.9	55	11.2	44	9.0	20.28	< 0.001*
More than one	2	10.0	12	60.0	3	15.0	0	0.0	1	5.0	2	10.0		
Sexual experience														
Yes	111	27.6	84	20.9	68	16.9	41	10.2	53	13.2	45	11.2	25.69	< 0.001*
No	338	36.6	123	13.3	147	15.9	136	14.7	107	11.6	72	7.8		
Sexual experience with current boyfriend/girlfriend														
Yes	93	28.7	64	19.8	56	17.3	37	11.4	42	13.0	32	9.9	6.98	0.222
No	18	23.1	20	25.6	12	15.4	4	5.1	11	14.1	13	16.7		
Sexual experience with one-night stand														
Yes	46	27.2	36	21.3	28	16.6	25	14.8	20	11.8	14	8.3	8.70	0.122
No	65	27.9	48	20.6	40	17.2	16	6.9	33	14.2	31	13.3		
Sexual experience with a prostitute 1 year prior to the study														
Yes	33	30.6	22	20.4	15	13.9	18	16.7	9	8.3	11	10.2	10.10	0.072
No	78	26.5	62	21.1	53	18.0	23	7.8	44	15.0	34	11.6		
Having a regular partner														
Yes	42	29.0	34	23.4	26	17.9	12	8.3	17	11.7	14	9.7	2.66	0.751
No	69	26.8	50	19.5	42	16.3	29	11.3	36	14.0	31	12.1		
Used alcohol or drugs prior to having sexual intercourse														
Yes	18	23.7	16	21.1	12	15.8	9	11.8	7	9.2	14	18.4	9.46	0.489
Sometime	5	27.8	2	11.1	2	11.1	3	16.7	3	16.7	3	16.7		
No	88	28.6	66	21.4	54	17.5	29	9.4	43	14.0	28	9.1		
HIV testing experience														
Yes	13	20.6	18	28.6	13	20.6	0	0.0	11	17.5	8	12.7	12.84	0.025*
No	98	28.9	66	19.5	55	16.2	41	12.1	42	12.4	37	10.9		
HIV testing 6 months prior to the study														
VYes	3	7.0	21	48.8	11	25.6	0	0.0	5	11.6	3	7.0	33.31	< 0.001*
No	108	30.1	63	17.5	57	15.9	41	11.4	48	13.4	42	11.7		
HIV testing 6 months to 1 year prior to the study														
Yes	4	9.8	15	36.6	12	29.3	0	0.0	6	14.6	4	9.8	19.58	0.001*
No	107	29.6	69	19.1	56	15.5	41	11.4	47	13.0	41	11.4		

^*^Significance level at α =0.05

According to the sexual patterns of males, 11.5% were MSM, and 4.6% were bisexual. For females, 5.0% preferred to have sex with females, and 12.0% were bisexual. One-third met their boyfriend or girlfriend through Facebook, and another one-third met them as classmates at school. Among those who had sexual experience, 88.1% reported having their first intercourse with a boyfriend or girlfriend, and more than half did not use a condom. Almost one-third (26.9%) of participants had sex with a prostitute 1 year prior to the study, and 8.0% engaged in commercial sex (Table 6).

**Table 6:** Sexual patterns among the participants who had sexual experience

Characteristics	n	%
Total	1325	100.0
Male sexual orientation (*n* = 524)		
Males who have sex with males (MSM)	60	11.5
Males who have sex with females	440	84.0
Males who have sex with both males and females (Bisexual)	24	4.6
Female sexual orientation (*n* = 801)		
Females who have sex with males	665	83.0
Females who have sex with females	40	5.0
Females who have sex with both males and females	96	12.0
Channel of meeting boyfriend/girlfriend		
Facebook	172	33.8
Line	2	0.4
Phone	40	7.9
Night club	14	2.8
Work together	12	2.4
Classmate	171	33.6
Live in the same village	98	19.3
First sexual intercourse with (*n* = 402)		
Boyfriend/girlfriend	354	88.1
Spouse	41	10.2
Prostitute	2	0.5
One-night stand	5	1.2
Used condom while having first sexual intercourse (*n* = 402)		
Yes	193	48.0
No	190	57.2
Cannot remember	19	4.8
Had a sexual experience as a male having sex with males (*n* = 181)		
Yes	52	28.8
No	129	71.2
Had sex with a prostitute 1 year prior to the study (*n* = 402)		
Yes	108	26.9
No	294	73.1
Participated in sexual swinging (*n* = 402)		
Yes	7	1.7
No	338	84.1
No answer	57	14.2
Worked as a sex worker (*n* = 402)		
Yes	32	8.0
No	370	92.0
Had a sexual experience with a foreigner (*n* = 402)		
Yes	37	9.2
No	365	90.8

Regarding STIs, 78.1% of females had known STIs, 47.7% had pelvic pain, 8.6% had a bad smell, 0.2% had discharge, 1.2% had vaginal ulcers, and 1.7% had lymphadenitis in the groin area. In males, 63.5% had known STIs, 3.4% had discharge, 12.0% had pain during urination, and 4.4% had lymphadenitis in the groin area. There was a significant difference in the proportion of those who had known STIs, discharge (a condition in which fluid drained from the genital area as a result of a bacterial, viral or yeast infection), pain during urination, and lymphadenitis in the groin area between both sexes (Table 7).

**Table 7:** STI symptoms among the participants

Characteristics	Female (Total = 801)		Male (Total = 524)		χ^2^	*p*-value
	n	%	n	%		
Known STIs						
Yes	626	78.1	333	63.5	40.89	< 0.001^*^
No	100	12.5	135	25.8		
Not sure^b^	75	9.4	56	10.7		
Pelvic pain						
Yes	382	47.7	N/A	N/A	N/A	N/A
No	340	42.4	N/A	N/A	N/A	N/A
Not sure^b^	79	9.9	N/A	N/A	N/A	N/A
Discharge						
Yes	2	0.2	18	3.4	22.61	< 0.001^*,a^
No	773	96.6	470	89.7		
Not sure^b^	26	3.2	36	6.9		
Bad smell from vagina						
Yes	69	8.6	N/A	N/A	N/A	N/A
No	642	80.1	N/A	N/A	N/A	N/A
Not sure^b^	90	11.3	N/A	N/A	N/A	N/A
Pain during urination						
Yes	51	6.4	63	12.0	14.93	< 0.001^*^
No	700	87.4	407	77.7		
Not sure^b^	50	6.2	54	10.3		
Vaginal ulcer						
Yes	10	1.2	N/A	N/A	N/A	N/A
No	759	94.8	N/A	N/A	N/A	N/A
Not sure^b^	32	4.0	N/A	N/A	N/A	N/A
Lymphadenitis in groin area						
Yes	14	1.7	23	4.4	9.31	< 0.002^*^
No	756	94.4	452	86.3		
Not sure^b^	31	3.9	49	9.3		
Vaginal itching						
Yes	109	13.6	N/A	N/A	N/A	N/A
No	601	75.0	N/A	N/A	N/A	N/A
Not sure^b^	91	11.4	N/A	N/A	N/A	N/A

^*^Significance level at α =0.05

^a^Fisher exact test

^b^These row numbers were not included in the analysis

From a total of 1325 participants, 836 blood specimens (63.1%) were voluntarily obtained. Among those who allowed blood specimen collection, nobody was positive for anti-HIV-I and -II, 6.4% were positive for anti-HBs, 1.9% were positive for HBsAg, and 0.2% were positive for anti-HCV. Only 3 participants (3.5%) reported having been vaccinated for hepatitis B and were positive for anti-HBs (Table 8).

**Table 8:** Prevalence of human immunodeficiency virus antibody-I and-II, hepatitis B surface antibody, hepatitis B surface antigen, hepatitis C antibody among the participants

Characteristics	n (Total = 836)	%
Human immunodeficiency virus antibody- I and-II		
Positive	0	0.0
Negative	836	100.0
Hepatitis B surface antibody		
Positive	85	10.2
Negative	751	89.8
Hepatitis B surface antigen		
Positive	25	2.9
Negative	811	97.1
Hepatitis C antibody		
Positive	2	0.2
Negative	834	99.8

## Discussion

Several risk behaviors and sexual behaviors related to HIV, HBV, and HCV infections were detected among hill tribe youths in Thailand, such as having number of boyfriends or girlfriends, having sexual experience, sexual experience with one-night stands, sexual experience with a prostitute within 1 year prior to the study, etc. A large proportion of hill tribe youths have pierced ears, use alcohol, use a shared toothbrush, have work experience outside the village, smoke and have tattoos. However, less than 10.0% had a history of hepatitis B vaccination. Regarding sexual experience, almost one-third had boyfriends or girlfriends, and most of them had already had sexual intercourse with their partners. A large proportion had sexual experience with one-night stands and prostitutes with 1 year prior to the study. Only 15.7% had a history of HIV testing.

The seroprevalence of HIV and HCV was low; however, the prevalence of HBV was high (2.9% for HBsAg and 10.2% for anti-HBs). UNAIDS reported that in 2017, the HIV prevalence among people aged 15 and older was 0.2–0.4% [41]. However, in our study, among 836 blood specimens obtained from the participants, none were positive for HIV. Leroi et al. [42] reported that the seroprevalence of HBsAg in Thailand was 5.1%, which is greater than that found in our study. In a recent study on the seroprevalence of HCV in the Thai population, Wasitthankasem, et al. [43] reported a seroprevalence of 0.9%, which was greater than that in the hill tribe youths from our study at 0.2%.

Several studies have reported the impact of sociocultural factors on risk behaviors and sexual behaviors in different communities [5, 10–12]. A study on culture and sexual behaviors among Iranian and New Zealand women found that sexual behaviors and sexual desire and pleasure were related to community culture, beliefs and past experiences regarding sexual activities [44]. A change in the social context or diversity of socialization led to a change in risky behaviors and sexual behaviors among youths in Ghana [45]. Moreover, a study by Mpondo et al. [46] reported that apart from culture, parenting styles and school curriculum affected risk behaviors, including sexual behaviors, of youths. A study in Nordic countries demonstrated that cultural and socioeconomic factors were associated with substance use among youths [47]. This was supported by a study in the United States that was conducted in different minority high school children with different cultural backgrounds, and it demonstrated that there were different risk behaviors, including sexual behaviors, particularly related to HIV infection [48]. This finding coincides with our study, which found that some behaviors, including sexual behaviors, are different between sexes and tribes, such as ear piercing, alcohol use, smoking, sexual experience, number of sexual partners, etc., which might be affected by the participants’ cultures and socioeconomic statuses. For instance, Akha people accept polygamy, while ear piercing is common in Lisu females [35, 49]. Moreover, Lahu males accept starting drinking alcohol in young age [50].

Many studies conducted in different countries, regions, and populations have demonstrated the associations of risk behaviors, sexual behaviors, and HIV, HBV, and HCV prevalence among youths [8, 14, 18]. They clearly showed that several risk behaviors and sexual behaviors impacted individual and community health in both developed and developing countries. A study of youths’ risky behaviors in the United States in 2017 showed that sex, race/ethnicity, and number of partners, including substance use among youths, were associated with youths’ health, particularly regarding HIV and HBV infections [51]. A systematic review on the prevalence of drug use, sexual activities, tattooing, and piercing from 2007 to 2017 included 9303 publications from around the world and reported that these behaviors were associated with HIV, HBV, and HCV [52]. A study in Nigeria reported that sharing items, tattooing, and a history of surgery were highly associated with HBV infection [53]. Ssewanyana et al. [54] conducted a systematic review and meta-analysis on health risk behaviors among adolescents in sub-Saharan Africa and found that the adolescents in the area had unprotected sexual intercourse with boyfriends or girlfriends leading to HIV and HBV infection. Moreover, a study in Beijing, China in 2018 reported that illegal drug use, number of sexual partners, and condom use were predictors of HIV infection among MSM aged 15–14 years [55]. Tran et al. [56] also reported that substance use and sexual intercourse with sex workers were associated with HIV infection among ethnic minority youths in Vietnam. In addition, a study in China by Pei et al. [57] on rural ethnic minority youths found that working outside the village and illiteracy were risk behaviors associated with HIV infection. In our study, there were several risk behaviors and sexual behaviors for HIV, HBV, and HCV infections among the hill tribe youths in Thailand, such as ear piercing, using alcohol, using a shared toothbrush, working outside the village, smoking, tattooing, sexual experience at an early age, and having a large number of sexual partners.

One interesting finding from our study was that the hill tribe youths in Thailand were highly vulnerable to HIV, HBV, and HCV infections due to their own behaviors, poor health education, low socioeconomic status, exposure to people outside the village, and low coverage of vaccination. In addition, the degree of behavioral expression and sexual behavior among the tribes and between the sexes were also different. This means that the different sexes and different tribes have different levels of exposure to HIV, HBV, and HBV. This coincides with some studies conducted in Thailand [20, 21, 24].

Some limitations were found in this study that could impact the analysis of the findings. Of those who participated in the study, 36.9% were not willing to provide blood specimens for HIV, HBV, and HCV marker detection. Two characteristics were significantly different between those who submitted blood specimens and those who did not: sex and tribe (see Appendix). Females were more willing to submit blood specimens than males, and Akha and Lahu participants were less willing to submit blood specimens than the others. However, the remaining number of participants who voluntarily allowed their blood specimens to be obtained was enough to be representative of the seroprevalence of HIV, HBV, and HCV among hill tribe youths in Thailand. Another factor that could impact the results is recall bias, such as the questions on number of partners, history of HBV vaccination, etc. An advantage of the cross-sectional design, which demonstrated the seroprevalence of the diseases, is that the results can be used in public health policy development, especially for resource allocation. This study also provided information on risk behaviors and sexual behaviors among the minority who are highly vulnerable to HIV, HBV, and HCV infections, which could be used for developing effective public health interventions for certain populations in the future.

## Conclusions

A large proportion of the hill tribe youths in Thailand engage in various risk behaviors and sexual behaviors related to HIV, HBV and HCV infections, such as substance use, using a shared toothbrush, injection with illegal substances, and working outside the village. Additionally, they had a low rate of HBV vaccination and a high rate of ear piercing, which had a significantly high prevalence rate in females. Hill tribe youths are also exposed to sexual experience at an early age, many sexual partners, prostitutes, and one-night stands, but most of them are not regularly tested for HIV. Under the conditions of economic constraints and poor education, many hill tribe youths desire to work outside the community and improve their families’ economic status; as such, they become a new vulnerable population for HBV, HCV and HIV infections. Providing a national-specific policy and strategy to improve the school attendance rate and create jobs with reasonable wages for hill tribe youths is urgently required to prepare and maintain them in their home setting, while effective health education and risk and sexual behavior change interventions are also needed.

## Data Availability

Since some of the variables are sensitive, the raw data supporting these findings are available by personal request at Tawatchai.api@mfu.ac.th

## Appendix

**Table 9:** Comparison of key characteristics between those who submitted blood specimens and those who did not

Characteristics	Obtained blood (Total = 836)		No obtained blood (Total = 489)		χ^2^	*p*-value
	n	%	n	%		
Sex						
Male	312	59.5	212	40.5	4.69	0.030^*^
Female	524	65.4	277	34.6		
Age (year)						
15–17	492	63.1	288	36.9	0.41	0.813
18–20	248	62.3	150	37.7		
21–24	96	65.3	51	34.7		
Tribe						
Akha	251	55.9	198	44.1	56.42	< 0.001^*^
Lahu	110	53.1	97	46.9		
Hmong	132	61.4	83	38.6		
Yao	136	76.8	41	23.2		
Karen	130	81.3	30	18.8		
Lisu	77	65.8	40	34.2		
Sexual experience						
Yes	243	60.4	159	39.6	1.73	0.188
No	593	64.2	330	35.8		
Known STIs						
Yes	602	62.7	357	37.3	0.08	0.771
No	156	66.4	79	33.6		
Not sure	78	59.5	53	40.5		
Alcohol use						
Yes	105	63.3	61	36.7	2.15	0.341
Ever	75	57.3	56	42.7		
No	656	63.8	372	36.2		
Smoking						
Yes	70	59.8	47	40.2	4.07	0.130
Ever	40	53.3	35	46.7		
No	726	64.1	407	35.9		

^*^Significance level at α =0.05

## Abbreviations

AIDS: Acquired immunodeficiency syndrome
Anti-HBs: Antibody to hepatitis B surface antigen
Anti-HCV: Antibody to hepatitis C virus
Anti-HIVI&II: Antibody to human immunodeficiency I&II
HBsAg: Hepatitis B surface antigen
HBV: Hepatitis B virus
HCV: Hepatitis C virus
HIV: Human immunodeficiency virus
ID: Identification card
IOC: Item-objective congruence technique
MSM: Men who have sex with men
NPV: Negative predictive value
STIs: Sexually transmitted infections
WHO: World Health Organization
